# Ancient and dominant: a novel feline immunodeficiency virus subtype “*X-EGY*” identified in Egyptian cats associated with high prevalence

**DOI:** 10.1186/s12917-025-04943-1

**Published:** 2025-07-29

**Authors:** Mahmoud S. Safwat, Amany D. Bahr, Noha M. Bakry, Haitham M. Amer, Ausama A. Yousif, Amir A. Shehata, Othman N. O. Mansour, Nehal M. Shahen, Reham Karam, Samah Eid, Ghada M. Khalil, Omnia H. Refaei

**Affiliations:** 1 Department of Internal Medicine and Infectious Diseases (Infectious Diseases), Faculty of Veterinary Medicine, Cairo University, Giza, 12211 Egypt; 2 Department of Internal Medicine and Infectious Diseases (Infectious Diseases), School of Veterinary Medicine, Badr University in Cairo, Cairo, Badr City, 11829 Egypt; 3 Department of Virology, Faculty of Veterinary Medicine, Cairo University, Giza, 12211 Egypt; 4 Genome Research Unit (GRU), Animal Health Research Institute (AHRI), Dokki, Giza 12618 Egypt; 5 Virology Research Department, Animal Health Research Institute (AHRI), Dokki, Giza 12618 Egypt; 6 Department of Virology, Faculty of Veterinary Medicine, Mansoura University, Mansoura, 35511 Egypt; 7 WEQAA Central Laboratory, National Centre for the Prevention and Control of Plant and Animal Diseases (WEQAA), Riyadh, 11454 Saudi Arabia; 8 Department of Bacteriology, Animal Health Research Institute (AHRI), Dokki, Giza 12618 Egypt; 9 Department of Clinical Pathology, Faculty of Veterinary Medicine, Cairo University, Giza, 12211 Egypt

**Keywords:** Clade, clinicopathological abnormalities, Egypt, Feline immunodeficiency virus, Molecular characterization, Prevalence, Phylogenetic analysis, Subtypes

## Abstract

**Background:**

Data on the epidemiology and molecular characterization of feline immunodeficiency virus (FIV) in Egypt are limited. This study aimed to estimate FIV prevalence in 240 Egyptian cats during 2022–2024 using three diagnostic techniques: two point-of-care antibody detection kits (Anigen^®^ and SNAP^®^) and one end-point PCR targeting the *env* gene. FIV infection is defined as positivity in at least two of the three diagnostic methods or PCR alone confirmed by sequencing, Additionally, FIV-associated clinicopathological abnormalities were assessed, and, for the first time in Egypt, circulating FIV subtypes were identified through partial sequencing and phylogenetic analysis of all *env* gene-positive samples (*n* = 10), along with 4 additional *gag* gene-positive samples.

**Results:**

Using our diagnostic criteria, 76 of 240 cats (31.7%) were identified as FIV-infected. Of these 76 cases, 75 were positive on both rapid kits, yielding a sensitivity of 98.7% for sequential testing with Anigen^®^ and SNAP^®^, whereas only 10 were positive on PCR and sequencing (13.2% sensitivity). FIV-infected cats exhibited lymphopenia, thrombocytosis, hyperglobulinemia, and reduced albumin/globulin ratios. On *env* and *gag* gene-based phylogenetic analyses, Egyptian strains did not cluster with any known FIV subtype (A-F and U-NZ*env*) but formed a distinct, previously uncharacterized clade. The Egyptian *env* sequences displayed low intra-group diversity (2.8–3.7%) but high divergence from all known subtypes (21–25%), with no evidence of recombination observed. Moreover, these *env* sequences were derived from both shelter-housed and client-owned cats across three Egyptian governorates within a one-year period.

**Conclusion:**

Given their genetic distinctiveness and widespread detection, we propose a novel FIV subtype, tentatively designated “X-EGY.” Its dominance and limited variability among its strains suggest it represents an ancient lineage uniquely adapted to Egyptian cats, rather than a recently emerged variant. This subtype may partly contribute to Egypt’s notably high FIV prevalence. Serological testing, utilizing two point-of-care kits in screening and confirmation steps, is the most accurate FIV diagnostic approach, outperforming molecular testing, particularly in regions where genetic data on circulating strains are scarce. Overall, the findings enhance our understanding of FIV epidemiology and diagnostic strategies and offer new insights into viral diversity and evolution.

## Background

Feline immunodeficiency virus (FIV) is a well-known feline pathogen that may cause severe immunosuppression in cats [1]. FIV belongs to the genus *Lentivirus* within the family *Retroviridae* and has a single-stranded RNA genome of approximately 9.4 kb that consists of three major genes: *gag*, *pol*, and *env*, encoding the capsid proteins, viral enzymes, and envelope proteins, respectively [2].

FIV is classified into seven distinct subtypes (A-F and U-NZ*env*) based on phylogenetic analyses of the highly variable regions 3–5 of the *env* gene (*env3-5*), with average intra- and inter-subtype divergence rates of 2.5–15% and 17–26%, respectively [3–6]. Similar subtyping results have been obtained using the variable regions 3–4 of the *env* gene (*env3-4*) and *gag* genes [5, 7]. Subtypes A, B, and C are the predominant ones identified in several countries worldwide; in contrast, subtypes D, E, F, and U-NZ*env* are localized in Japan, Argentina, Portugal, and New Zealand, respectively [2, 5].

The saliva of infected cats typically contains high viral loads, and transmission occurs through biting; consequently, the disease is more prevalent in cats who are males, adult, sexually intact, exhibiting aggressive behavior, having an outdoor access lifestyle, and/or living in multicat environments such as shelters [8]. FIV has a global distribution, with the lower (3.8%) and higher (21.6%) mean prevalence rates recorded in North America and Africa, respectively [9].

FIV infection in cats typically progresses through three phases: acute, subclinical, and terminal [1]. In the terminal phase, FIV-infected cats may exhibit immunosuppression signs, including recurrent or chronic infections, neurological signs, lymphoma, mild to moderate anemia, lymphopenia, neutropenia, hypergammaglobulinemia, and a decreased albumin-to-globulin ratio [2, 10].

Routine diagnosis of FIV infection is primarily performed using point-of-care (PoC) kits and PCR, which detect virus-specific antibodies and integrated DNA (provirus), respectively [1]. However, the results of these assays vary depending on the stage of infection. In the acute phase, cats may test positive or negative depending on the interval between infection and testing, as proviral DNA loads and antibody titers become detectable only at 2–4 and 4–8 weeks post-infection, respectively. In the subclinical and terminal phases, cats are typically antibody-positive but may test positive or negative for PCR. In rare cases, during the terminal phase, cats may appear antibody-negative [11]. PoC kits can detect vaccine-induced and maternally derived antibodies (up to the age of six months), resulting in false-positive results [12, 13]. Notably, the only commercially available FIV vaccine, Fel-O-Vax^®^, is not marketed in Egypt and is currently available only in Oceania and Japan [11]. The lower diagnostic accuracy of PCR compared to PoC kits is primarily attributed to low proviral DNA loads and the high genetic diversity of this virus [11, 14].

In Egypt, data on FIV epidemiology are limited to a single study that investigated FIV in feral cats in Giza Governorate, recording one of the highest reported FIV prevalences worldwide (33.9%) [9, 15]. The disease burden in other cat populations, including client-owned and shelter-housed cats, remains to be elucidated. Moreover, data on subtypes and genetic diversity of FIV Egyptian strains are lacking. Therefore, this study aimed to: (1) estimate the prevalence of FIV infection in a random sample of client-owned and shelter-housed cats in Egypt using combined serological and molecular diagnostics; (2) assess clinicopathological changes associated with FIV infection using an inclusive approach that reflects natural field variability; and (3) genetically characterize FIV-positive samples, for the first time in Egypt, through partial *env* and *gag* sequence analysis for subtype classification, divergence estimation, recombination screening, and amino acid variation.

## Materials and methods

### Sample size calculation, study population, and blood sampling

The number of cats required to estimate FIV prevalence in Egypt on a statistical basis was calculated to be 240 using the following formula: $\:n=\frac{{Z}^{2}Pexp(1\:-\:Pexp)}{d^2}$ [16], with the following parameter values: 90% confidence level (*Z* = 1.645), 5% precision (*d* = 0.05), and 33.9% expected prevalence (*Pexp* = 0.339) [15].

The characteristics of the study population in this research were previously detailed in a recent survey on hemotropic mycoplasma species in Egyptian cats conducted in our laboratory [17]. In brief, 240 cats were randomly selected from three Egyptian governorates: Cairo (*n* = 112), Giza (*n* = 113), and Al-Qalyubia (*n* = 15), between December 2022 and April 2024. The recruited cats included both client-owned (*n* = 46) and shelter-housed (*n* = 194) individuals, ranging in age from eight months to fifteen years (median age = six years). The population comprised 140 males (55 intact and 85 castrated) and 100 females (80 intact and 20 spayed). Clinical status was assessed at the time of sampling through physical examination and review of medical records.

All randomly selected cats were included in the clinicopathological analysis without applying additional inclusion or exclusion criteria in order to reflect the natural diversity of FIV infection. EDTA-whole blood samples (4 ml) were collected from all cats to detect FIV-specific antibodies and DNA and investigate FIV-associated hematological abnormalities. Additional blood samples (1 ml) were collected into plain tubes to assess FIV-associated serum biochemical abnormalities. The Institutional Animal Care and Use Committee (IACUC) at the Faculty of Veterinary Medicine, Cairo University, approved the current study (Vet CU 03162023753). Oral informed consent was obtained from the guardians of client-owned cats and shelter directors before sample collection and data use.

### Screening of blood samples for FIV infection

The FIV infection status of all 240 recruited cats was investigated using a diagnostic panel, consisting of three diagnostic techniques (two point-of-care antibody detection kits and one PCR assay). PoC kits used in this study were Anigen^®^ FIV Ab/FeLV Ag (Bionote, Korea) and the SNAP^®^ Combo FIV Ab/FeLV Ag (IDEXX, USA) kits. PoC kits were performed on fresh EDTA whole blood samples, as recommended by the manufacturers. According to manufacturers, the Anigen^®^ kit has 100% sensitivity and specificity, while the SNAP^®^ kit shows 93.5% sensitivity and 100% specificity. Independent studies in nonvaccinated cats report 88.9–100% sensitivity and 99–100% specificity for Anigen^®^, and 88.9–100% sensitivity and 97–100% specificity for SNAP^®^ [18–20]. The SNAP^®^ Combo test detects antibodies against *gag*-encoded proteins, including matrix (p15) and capsid (p24), whereas the Anigen^®^ Rapid test targets antibodies to the transmembrane envelope protein (gp40) [21]. However, the specific FIV subtype(s) from which these antigens were derived remain undisclosed by the manufacturers and, to our knowledge, are not reported in the scientific literature.

Molecular testing was performed to detect FIV-specific proviral DNA using one end-point PCR assay; total DNA was extracted from blood samples (200 µl) using the QIAamp DNA Blood Mini Kit (Qiagen, Hilden, Germany) according to the manufacturer’s protocol. FIV proviral DNA was identified by amplifying the *env*3-5 gene using the DreamTaq PCR Master Mix (ThermoFisher, USA) and the primer pairs FIV7224 for: 5′-GTACAGACCCATTACAAATC-3′ and FIV8000rev: 5′-CTGCCACTGGGTTATACCAA-3′ [22, 23]. PCR amplification was performed in a GeneAmp 9700 thermal cycler (ThermoScientific, USA) under the following cycling conditions: initial denaturation at 94 °C for 5 min; 35 cycles of denaturation at 94 °C for 30 s, annealing at 54 °C for 30 s, and extension at 72 °C for 50 s; followed by a final extension step at 72 °C for 10 min. Positive reactions were concluded by visualization of specific amplicons at the expected band size (776 bp) in agarose gel.

Cats were classified as FIV-infected if they tested positive for at least two of the three diagnostic techniques [1, 11, 20]. One exception to this rule was that cats who tested positive only for PCR were considered FIV-infected if the identity of the detected amplicons was confirmed as FIV by sequencing [24].

### Hematological and serum biochemical investigations

Complete blood count (CBC), including red blood cell (RBC) count, hemoglobin (Hgb) concentration, hematocrit (HCT), and total leukocytic count (TLC), was evaluated using the ABC™ Animal Blood Counter (ABC Vet, France). Differential leukocyte counts were determined by microscopic examination of Diff-Quik-stained peripheral blood smears, while platelet counts were assessed using the direct manual dilution method with a hemocytometer.

Serum levels of total proteins (TP), albumin, total bilirubin (TBIL), creatinine, and alanine aminotransferase (ALT) were measured using a semi-automated biochemical analyzer based on spectrophotometric readings at specific wavelengths (Robonik, India) using specific kits (Spectrum Diagnostics, Egypt). Serum globulin levels were estimated by subtracting albumin from TP, and the albumin: globulin (A: G) ratio was subsequently calculated. Reference ranges of hematological and biochemical parameters are provided in Table 5 [25].

### Genetic analysis and subtyping of FIV-positive samples

All samples positive by *env*3-5 PCR were further subjected to another end-point PCR targeting the *gag* gene. This assay amplified a 675 bp fragment using primers FIV1026for (5′-GGCATATCCTATTCA AACAG-3′) and FIV1700rev (5′-AAGAGTTGCATTTTATATCC-3′) [22, 23] under the following cycling conditions: initial denaturation at 94 °C for 5 min, 45 cycles of denaturation at 94 °C for 45 s, annealing at 50 °C for 45 s, extension at 72 °C for 45 s, and a final extension at 72 °C for 7 min.

The amplicons of both *env3-5* and *gag* PCR were purified using the QIAquick^®^ Gel Extraction Kit (Qiagen) and sequenced on both strands using the BigDye Cycle Sequencing Kit (ThermoFisher), following the manufacturer’s instructions. Sequence contigs of *env3-5* and *gag* genes were edited and assembled using BioEdit software version 7.2.5.

FIV *env3-5* and *gag* gene sequences available in GenBank (accessed in December 2024) that matched the genomic position and nucleotide length (628 and 600 nt for *env3-5* and *gag* genes, respectively) of Egyptian sequences and represented different geographic areas, and periods were selected as reference sequences. However, these reference sequences represent only five subtypes (A-E). To include more reference FIV subtypes, another *env* (*env3-4*) and *gag* gene sequences present in GenBank that matched our strains but in shorter gene segments (480 and 300 nt for *env3-4* and *gag* genes, respectively) were also involved in the reference sequences dataset. Egyptian sequences were aligned with reference sequences using the “ClustalW” tool implemented in BioEdit software.

All evolutionary analyses were conducted using MEGA software version 11. The phylogenetic relationship between Egyptian and reference FIV strains was inferred for different gene segments using the maximum likelihood (ML) statistical method. Nucleotide substitution models that best fit our dataset were selected using the “Find Best DNA Model” tool implemented in MEGA, including the general time reversal model with a discrete gamma distribution and allowing for invariant sites (GTR + I + G) for both *env3-5* and *env3-4* gene segments and the Tamura-3-Parameter model with a discrete gamma distribution (T92 + G) for both the 600 nt and 300 nt *gag* gene segments. The branching order reliability of phylograms was assessed using the bootstrap method (1000 replicates). The obtained trees were edited using FigTree software version 1.4.4. The average genetic divergence percentage within and between different FIV subtypes for different *env* gene segments (*env3-5* and *env3-4*) was calculated using the Kimura-2-Parameter (K2P) pairwise genetic distance matrix. Recombinant analyses for different alignments were conducted using Recombinant Detection Program software version 5 (RDP5) with the following recombination detection methods: RDP, GENECONV, BootScan, MaxChi, Chimaera, SiScan, and 3Seq; recombination events detected at least by 4 algorithms at a *P* ≤ 0.01 were only considered.

Egyptian and reference *env3-5* and *env*3-4 nucleotide alignments were translated into deduced amino acid (aa) sequences. The evolutionary history of *env3-5* and *env3-4* aa sequences was inferred using the maximum likelihood method and the Jones-Taylor-Thornton model with a discrete gamma distribution and allowing for invariant sites (JTT + I + G) as the best aa substitution model that would fit our dataset. The average genetic divergence percentage within and between different FIV subtypes for *env3-5* aa and *env3-4* aa sequences was calculated from the JTT pairwise genetic distance matrix. A consensus sequence was generated for the *env*3-5 aa sequences alignment using UGENE software version 51.0; the conservation of cysteine residues among all *env3-5* aa sequences was evaluated, and *N*-linked glycosylation sites were predicted using the N-GlycoSite tool (https://www.hiv.lanl.gov/content/sequence/GLYCOSITE/glycosite.html) (accessed in March 2025) based on the following formula: N-X-S or N-X-T, where X can be any amino acid except proline.

### Statistical analysis

All statistical analyses were performed using SPSS software and online statistical tools (https://www.openepi.com and https://astatsa.com/FisherTest). FIV prevalence was calculated as the proportion of FIV-infected cats, determined by the diagnostic panel, among the study population. The FIV prevalence’s 95% confidence interval (CI) was calculated using the Wald normal approximation method.

The sensitivity of sequential serological testing using Anigen^®^ and SNAP^®^ was defined as the proportion of cats that tested positive on both kits among those classified as truly FIV-infected, based on the study’s diagnostic criteria. Similarly, the sensitivity of molecular testing was calculated as the proportion of truly infected cats that tested positive by PCR and were subsequently confirmed by sequencing.

The distribution normality of continuous CBC and serum biochemical variables was determined using the Kolmogorov-Smirnov method. Normally distributed variables were expressed as means ± standard deviations (SD) and compared between FIV-infected and non-infected cats for statistically significant differences using the independent t-test. Non-normally distributed variables were presented as medians and interquartile ranges (IQR) and compared between FIV-infected and non-infected cats for statistically significant differences using the Mann-Whitney test.

To assess potential associations with FIV infection, a range of categorical variables were analyzed using the chi-square test (for expected cell counts > 5) or Fisher’s exact test (for counts ≤ 5), as appropriate. These included demographic variables, cat source (shelter vs. private), sex (male vs. female), neuter status (intact vs. castrated for males; intact vs. spayed for females), and locality (Cairo, Giza, or Qalyubia), as well as clinicopathological parameters (CBC and serum biochemical).

To minimize the risk of Type I error due to multiple comparisons, a Bonferroni correction was applied by dividing 0.05 by the number of variables tested (15 continuous and 20 categorical) [26], resulting in adjusted significance thresholds of *P* < 0.0033 for continuous variables and *P* < 0.0025 for categorical variables. Odds ratios (ORs) with 95% confidence intervals (CIs) were calculated for categorical variables showing statistically significant associations.

## Results

### FIV infection status, risk factors, and recorded medical conditions

Of the 240 cats examined in this study, 78 (32.5%) and 76 (31.7%) tested positive using Anigen^®^ and SNAP^®^ kits, respectively. In contrast, only 10 cats (4.2%) were positive by the *env*3-5 PCR methodology, and all these PCR-positive samples were confirmed by sequencing.

When combining the results from all three diagnostic techniques (Table 1): (a) nine cats (9/240, 3.8%) tested positive by all three methods; (b) sixty-six cats (66/240, 27.5%) were positive by only two methods, all of which were positive by both the Anigen^®^ and SNAP^®^ kits, with no cats testing positive by PCR and only one of the serological kits; (c) five cats (5/240, 2.1%) were positive by only one method, including one cat positive by PCR alone, three cats positive by Anigen^®^ alone, and one cat positive by SNAP^®^ alone; and (d) the remaining 160 cats (160/240, 66.7%) were negative by all three diagnostic methods.

**Table 1:** Summary of all possible result combinations from the three FIV diagnostic techniques used in this study, including Anigen^®^ and SNAP^®^ antibody detection kits, and one end-point PCR assay targeting *env*3-5, with corresponding FIV infection status based on the study’s diagnostic criteria

All possible combined results of all three diagnostic techniques used in this study				FIV infection status according to our study’s diagnostic criteria ^a^	Number of cats in this study
	Anigen^®^ Kit	SNAP^®^ kit	*env*3-5 PCR		
Positive for all diagnostic techniques	**+**	**+**	**+**	FIV-infected	9
Positive for two diagnostic techniques	**+**	**+**	**–**	FIV-infected	66
	**+**	**–**	**+**	FIV-infected	0
	**–**	**+**	**+**	FIV-infected	0
Positive for one diagnostic technique	**–**	**–**	**+** ^b^	FIV-infected	1
	**+**	**–**	**–**	FIV non-infected	3
	**–**	**+**	**–**	FIV non-infected	1
Negative for all diagnostic techniques	**–**	**–**	**–**	FIV non-infected	160

Cats were classified as FIV-infected if they tested positive for at least two diagnostic techniques, or only PCR with sequencing confirmation

^b^ PCR positivity was confirmed by sequencing

According to the combined results obtained and diagnostic criteria adopted in this study, the FIV prevalence in the study population was calculated as 76/240 (31.7%)

Based on our study’s diagnostic criteria described in the Materials and Methods section, 76 cats (76/240, 31.7%) were classified as truly FIV-infected. Accordingly, the FIV prevalence in the study population was 31.7% ( 95% CI: 25.78–37.55%). The remaining 164 cats (164/240, 68.3%) were considered FIV non-infected.

Among the 76 truly FIV-infected cats, 75 were positive by both Anigen^®^ and SNAP^®^, yielding a sensitivity of sequential serological testing with Anigen^®^ and SNAP^®^ of 98.1%, while 10 cats were positive by PCR and sequencing, resulting in a sensitivity of molecular testing of 13.2%.

Among the demographic variables analyzed, cat source was the only factor significantly associated with FIV infection in the study population: shelter-housed cats had 4.73 times higher odds of FIV infection compared to client-owned cats (*P* < 0.001) (Table 2).

**Table 2:** Association of FIV infection with selected demographic variables

Variable	Categories	*n*	FIV-infected		*P* value ^a^	OR (95% CI)
			*N*	%		
Cat source	Shelter	194	71	37.4	**< 0.001**	4.73 (1.78–12.52)
	Private clinics	46	5	10		
Region	Cairo	112	38	33.9	0.08	-
	Giza	113	37	32.7		
	Al-Qalyubia	15	1	6.7		
Sex	Males	140	51	36.4	0.06	-
	Females	100	25	25		
Neuter status	Intact	55	22	40	0.48	-
	Castrated	85	29	34.1		
	Males					
	Intact	80	19	23.7	0.56	-
	Spayed	20	6	30		
	Females					

^**a**^ Categorical demographic and clinical variables were considered significantly associated with FIV infection at a P value < 0.0025, following Bonferroni correction. Accordingly, cat source was the only demographic variable that showed a statistically significant association with FIV infection (*P* < 0.001), as indicated in bold

Abbreviations (CI: confidence interval; FIV: feline immunodeficiency virus; OR: odds ratio)

Of the 76 FIV-infected cats, 36 (47.4%) were clinically healthy, while the remaining 40 (52.6%) presented with one or more medical conditions, including chronic upper respiratory infection (27/76, 35.5%), dermatophytosis (10/76, 13.2%), feline infectious anemia (8/76, 10.5%), and feline chronic gingivostomatitis (3/76, 3.9%). In comparison, among the 164 FIV-uninfected cats, 98 (59.8%) appeared clinically healthy, whereas 66 (40.2%) exhibited a range of clinical abnormalities. These included chronic upper respiratory infection (44/164, 26.8%), feline infectious anemia (9/164, 5.5%), and dermatophytosis (7/164, 4.3%). Additional diagnoses, each observed in a single individual (1/164, 0.6%), were feline chronic gingivostomatitis, acute gastroenteritis, chronic kidney injury, urolithiasis, aortic thromboembolism, and ulcerative keratitis.

### FIV-associated hematological and serum biochemical abnormalities

The differences in continuous variables between FIV-infected and non-infected cat groups are presented in Table 3. HCT%, lymphocyte counts, and A: G ratios were significantly lower in the FIV-infected group compared to the non-infected one. Notably, in both groups, the A: G ratios were below the reference value (> 0.8), while HCT% and lymphocyte counts remained within the reference ranges. The FIV-infected group also exhibited significantly higher TP and globulin concentrations, both exceeding the reference ranges, compared to the non-infected group. Interestingly, the globulin concentrations were also above the reference range in the non-infected group.

**Table 3:** CBC and serum biochemical continuous variables analysis of FIV-infected and non-infected cats

Parameters (measurement unit)	Reference range^a^	FIV-infected cats		FIV non-infected cats		*P* ͩ
		*n*	Mean ± SD^b^ or Median (IQR) ͨ	*n*	Mean ± SD^b^ or Median (IQR) ͨ	
HCT (%)	30–45	74	33 (27–37)	148	38 (31–42)	**< 0.001**
Hgb conc (g/dL)	9.8–15.4	74	10.96 ± 3.4	147	12.25 ± 3.47	0.009
RBCs (10⁶/µL)	5.0–10.0	74	8.4 (6.4–10.2)	147	9 (7.3–11.7)	0.04
PLT (10³/µL)	175–600	74	350 (250–675)	137	325 (250–450)	0.05
TLC (10³/µL)	5.5–19.5	74	15.8 (11.6–22.4)	147	15.6 (10.8–24.6)	0.69
NØ (10³/µL)	2.5–12.5	72	7 (3.6–11.8)	146	8.3 (5-12.2)	0.09
LØ (10³/µL)	1.5-7	72	2.1 (1-3.4)	146	3.3 (1.5–5.4)	**0.002**
MØ (10³/µL)	0-	72	0.57 (0.1–1.1)	146	0.58 (0.1–1.2)	0.95
TP (g/dL)	5.7–8.9	65	9.504 ± 1.62	85	7.952 ± 2.16	**< 0.001**
ALB (g/dL)	2.3–3.9	65	2.26 ± 0.26	85	2.25 ± 0.43	0.89
GLOB (g/dL)	2.8–5.1	65	7.23 ± 1.58	85	5.69 ± 1.99	**< 0.001**
A: G ratio	> 0.8	65	0.33 (0.26–0.36)	85	0.38 (0.32–0.51)	**< 0.001**
TBIL (mg/dL)	0-0.1	65	0.42 (0.18–1.2)	85	0.56 (0.24–0.96)	0.26
CREAT (mg/dL)	0.8–2.4	65	1.16 (0.95–1.48)	84	1.01 (0.81–1.28)	0.08
ALT (U/L)	12–130	64	16.8 (12-26.6)	85	22.2 (14-32.2)	0.06

Abbreviations (ALB: albumin; ALT: alanine aminotransferase; A: G ratio: Albumin: Globulin ratio; CBC: complete blood count; CREAT: creatinine; FIV: feline immunodeficiency virus; GLOB: globulin; HCT: hematocrit; Hgb: hemoglobin; IQR: interquartile range; LØ: lymphocyte; MØ: monocytes; NØ; neutrophils; PLT: platelets; RBCs: red blood cells; SD: standard deviation; TBIL: total bilirubin; TLC: total leucocyte count; TP: total proteins)

^a^ Reference [25] (Aiello and Moses, 2016)

^b^ Means ± SD were calculated for normally distributed CBC and serum biochemical continuous variables and were tested statistically by the independent t-test

ͨ Medians and IQR were calculated for non-normally distributed CBC and serum biochemical continuous variables and were tested statistically by the Mann-Whitney test

ͩ Following Bonferroni correction to eliminate multiple test interference, parameters with a *P* value of < 0.003 were considered statistically significant (shown in bold)

Table 4 summarizes the differences in categorical data between FIV-infected and non-infected groups. FIV infection was significantly associated with thrombocytosis, lymphopenia, hyperproteinemia, and hyperglobulinemia. Nonetheless, some FIV-infected cats exhibited thrombocytopenia and lymphocytosis.

**Table 4:** Comparison between FIV-infected and non-infected cats regarding categorical CBC and biochemical variables, including statistical significance

Parameter	Abnormality	FIV-infected^a^	FIV non-infected^a^	P^b^	OR (95% CI)
HCT	Decreased	28 (38%)	32 (22%)	0.01	-
RBCs	Decreased	9 (12%)	15 (10%)	0.658	-
Hgb	Decreased	27 (36.5%)	28 (19%)	0.004	-
**PLT**	Decreased	6 (8%)	11 (8%)	1.000	-
	**Increased**	22 (30%)	13 (9.5%)	**< 0.001**	4 (1.89–8.61)
TLC	Decreased	5 (6.75%)	8 (5.5%)	0.764	-
	Increased	26 (35%)	54 (37%)	0.814	-
NØ	Decreased	23 (32%)	34 (23%)	0.171	-
	Increased	4 (4.5%)	23 (15.5%)	0.032	-
**LØ**	**Decreased**	13 (18%)	7 (5%)	**0.001**	4.3 (1.6–11.5)
	Increased	15 (21%)	35 (24%)	0.57	-
MØ	Increased	22 (30.5%)	54 (37%)	0.348	-
**TP**	**Increased**	44 (67.5%)	27 (32%)	**< 0.001**	4.5 (2.25–8.99)
ALB	Decreased	33 (51%)	44 (52%)	0.902	
**GLOB**	**Increased**	61 (94%)	53 (62%)	**< 0.001**	9.2 (3-27.7)
TBIL	Increased	21 (32%)	22 (26%)	0.388	
CREAT	Increased	3 (5%)	1 (1%)	0.318	
ALT	Increased	0 (0%)	3 (3.5%)	0.259	

Abbreviations (ALB: albumin; ALT: alanine aminotransferase; CBC: complete blood count; CI: confidence interval; CREAT: creatinine; FIV: feline immunodeficiency virus; GLOB: globulin; HCT: hematocrit; Hgb: hemoglobin; LØ: lymphocyte; MØ: monocytes; NØ; neutrophils; OR: odds ratio; PLT: platelets; RBCs: red blood cells; TBIL: total bilirubin; TLC: total leucocyte count; TP: total proteins)

^a^The number of FIV-infected and non-infected cats tested for each variable is given in Table 3^b^Following Bonferroni correction to eliminate multiple test interference, parameters with a *P* value of < 0.003 were considered statistically significant (shown in bold)

### Genetic analysis of Egyptian FIV sequences

All ten *env*3–5 gene-based PCR-positive samples were successfully sequenced. Among them, only four (4/10, 40%) yielded strong *gag* gene PCR amplicons suitable for sequencing, most likely due to the long storage period before *gag* gene PCR testing. The GenBank accession numbers for the Egyptian *env* (*n* = 10) and *gag* (*n* = 4) gene sequences, along with the characteristics of the cats from which these sequences were derived, are provided in Table 5. All data regarding reference *env* and *gag* gene sequences retrieved from GenBank, including FIV subtype, strain name, country, GenBank accession number, and sequence length, are listed in Table 6.

**Table 5:** Egyptian FIV strains analyzed in this study: epidemiologic features and GenBank accession numbers

Strain name	Epidemiologic features					GenBank accession number	
	Age	Sex (neutering status)	Ownership type	Date of sampling	Governorate	Env gene	*gag* gene
14/EGY	3 years	Female (intact)	Client-owned	January 2023	Qalyubia	OR882079	PQ799461
26/EGY	11 years	Male (intact)	Client-owned	January 2023	Cairo	PQ799455	PQ799462
43/EGY	2 years	Male (intact)	Client-owned	March 2023	Giza	OR882080	-
102/EGY	5 years	Male (castrated)	Shelter-housed (Shelter 1)	January 2023	Giza	OR882081	-
200/EGY	7 years	Male (castrated)	Shelter-housed (Shelter 1)	June 2023	Giza	OR863244	-
202/EGY	10 years	Male (castrated)	Shelter-housed (Shelter 2)	July 2023	Cairo	PQ799456	-
223/EGY	6 years	Male (intact)	Shelter-housed (Shelter 3)	March 2024	Giza	PQ799457	-
239/EGY	4 years	Female (spayed)	Shelter-housed (Shelter 3)	March 2024	Giza	PQ799458	PQ799463
241/EGY	3 years	Male (intact)	Client-owned	April 2024	Cairo	PQ799459	-
244/EGY	5 years	Male (castrated)	Shelter-housed (shelter 3)	April 2024	Giza	PQ799460	PQ799464

**Table 6:** Strain’s name, country of origin, subtype, and GenBank accession numbers of reference FIV *env* and *gag* gene sequences used in this study

*env* gene sequences					*gag* gene sequences				
Strain name/Country	Subtype	Accession number	Nucleotide length (Variable regions)		Strain name/Country	Subtype	Accession number	Nucleotide length	
			628 nt (V3-5)	480 nt (V3-4)				600 nt	300 nt
Petaluma/USA	A	M25381	+	+	Petaluma/USA	A	M25381	+	+
TN7/CAN	A	GQ422134	+	+	TN7/CAN	A	DQ365594	+	+
GF6/COL	A	MN630242	+	+	GF6/COL	A	MN630242	+	+
Ca2/ZAF	A	DQ873714	+	+	Sendai1/JPN	A	D37820	+	+
Sendai1/JPN	A	D37813	+	+	17/CHN	A	MF352016	+	+
SAP01/JPN	A	AB010402	+	+	WO/FRA	A	L06136	+	+
17/CHN	A	MF352016	+	+	8/UK	A	GU055218	+	+
C18/CHN	A	KX646706	+	+	Z1/CHE	A	X57002	+	+
WO/FRA	A	L06312	+	+	BEANslbe/BEL	A	KM880116	+	+
4/NLD	A	X69498	+	+	BEWVdooo/BEL	A	KM880118	+	+
2/UK	A	X69494	+	+	USIL2489_7B/USA	B	U11820	+	+
8/UK	A	X69496	+	+	4B/BRA	B	MW142024	+	+
206,394/UK	A	HQ456827	+	+	34/BRA	B	MW142048	+	+
Z1/CHE	A	X57002	+	+	Pequeno/BRA	B	MF370550	+	+
USIL2489_7B/USA	B	U11820	+	+	M2/ITA	B	Y13867	+	+
Leviano_C8/BRA	B	FJ374695	+	+	M3/ITA	B	Y13866	+	+
4B/BRA	B	MW142024	+	+	TM2/JPN	B	M59418	+	+
34/BRA	B	MW142048	+	+	Sendai2/JPN	B	D37821	+	+
Pequeno/BRA	B	MF370550	+	+	Aomori2/JPN	B	D37824	+	+
LP9/ARG	B	D84497	+	+	C36/USA	C	AY600517	+	+
M2/ITA	B	X69501	+	+	BM3070/CAN	C	AF474246	+	+
M3/ITA	B	X69502	+	+	Shizuoka/JPN	D	AY679785	+	+
TM2/JPN	B	M59418	+	+	Fukuoka/JPN	D	D37822	+	+
NG4/JPN	B	GU066864	+	+	LP3/ARG	E	AB027302	**-**	+
AIC02/JPN	B	AB010397	+	+	LP20/ARG	E	AB027303	**-**	+
TY1/JPN	B	D67064	+	+	02/RUS	E	EF413007	**-**	+
Sendai2/JPN	B	D37814	+	+	06/RUS	E	EF413011	**-**	+
Aomori2/JPN	B	D37817	+	+	13/RUS	E	EF413005	**-**	+
CABCpbar01C/CAN	C	U02393	+	+	151_02LisP/PRT	F	DQ072543	**-**	+
C36/USA	C	AY600517	+	+					
BM3070/CAN	C	AF474246	+	+					
AIC01/JPN	C	AB010396	+	+					
2015/2/NZL	C	MW012628	+	+					
TI3/TWN	C	AB016027	+	+					
TI4/TWN	C	AB016028	+	+					
MU-1/TWN	C	AB016667	+	+					
VND-2/VNM	C	AB083503	+	+					
VND-7/VNM	C	AB083508	+	+					
Shizuoka/JPN	D	D37811	+	+					
Fukuoka/JPN	D	D37815	+	+					
KUM01/JPN	D	AB010398	+	+					
OKA01/JPN	D	AB010400	+	+					
MY2/JPN	D	D67062	+	+					
MC8/JPN	D	D67063	+	+					
VND-1/VNM	D	AB083502	+	+					
LP3/ARG	E	D84496	+	+					
LP20/ARG	E	D84498	+	+					
LP24/ARG	E	D84500	+	+					
151_02LisP/PRT	F	DQ072567	-	+					
EV01/NZL	U-NZ*env*	EF153969	-	+					
BS08/NZL	U-NZ*env*	EF153970	-	+					
TKP15/NZL	U-NZ*env*	EF153973	-	+					
TKP21/NZL	U-NZ*env*	EF153975	-	+					
MF19/NZL	U-NZ*env*	EF153976	-	+					
TKP88/NZL	U-NZ*env*	EF153977	-	+					
TKP22/NZL	U-NZ*env*	EF153979	-	+					
TKP94/NZL	U-NZ*env*	EF153980	-	+					
MF21/NZL	U-NZ*env*	EF153981	-	+					

(+) present in GenBank/ (-) absent in GenBank

The names of countries were abbreviated by 3-letter code as follows: ARG (Argentina), BEL (Belgium), BRA (Brazil), CAN (Canada), CHE (Switzerland), CHN (China), COL (Colombia), FRA (France), ITA (Italy), JPN (Japan), NLD (Netherlands), NZL (New Zealand), PRT (Portugal), RUS (Russia), TWN (Taiwan), UK (United Kingdom), USA (United States of America), VNM (Vietnam), ZAF (South Africa)

**Fig. 1:**
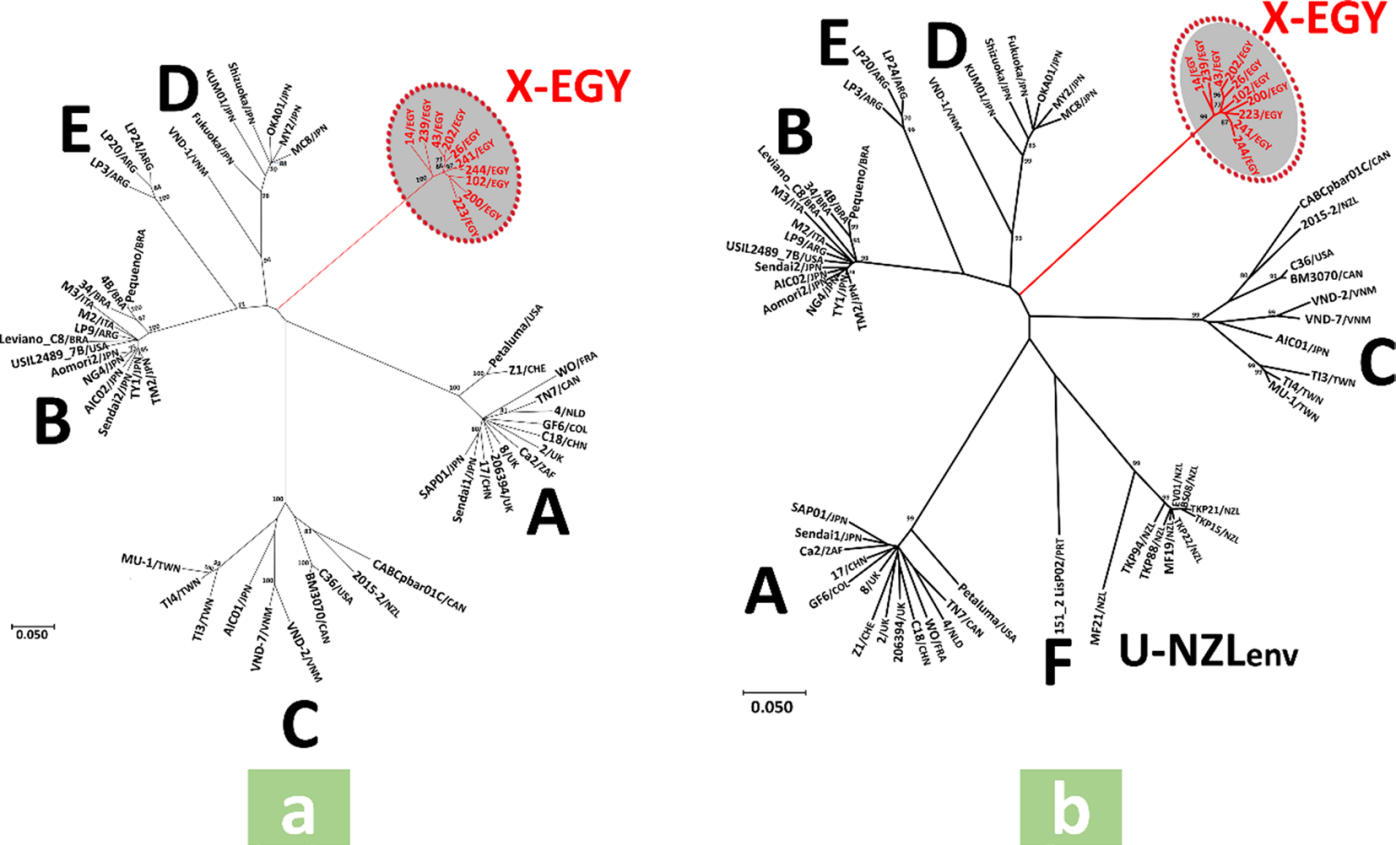
The *env* nucleotide sequence-based phylogenetic analyses conducted using the maximum likelihood statistical method and GTR + I + G nucleotide substitution model. The GenBank accession numbers of sequences used to construct these trees are given in Tables 5 and 6. Bootstrap values > 70% are displayed at branching nodes. Names of the previously characterized FIV subtypes are displayed next to corresponding clades. The Egyptian strains obtained in this study are marked by red font, and their previously uncharacterized clade, designated as “*X-EGY*”, is highlighted in grey; these 10 strains represent all samples that tested positive by end-point PCR targeting the *env*3–5 region among the 76 FIV-infected cats identified in this study (10/76, 13.2%). The scale bar represents the number of base substitutions per site. (**a**) The tree was constructed from 58 FIV nucleotide sequences encompassing the variable regions V3 to V5 of the *env* gene, and there were 628 positions in the final dataset. (**b**) The tree was constructed from 68 FIV nucleotide sequences encompassing the variable regions V3 to V4 of the *env* gene, and there were 480 positions in the final dataset

**Fig. 2:**
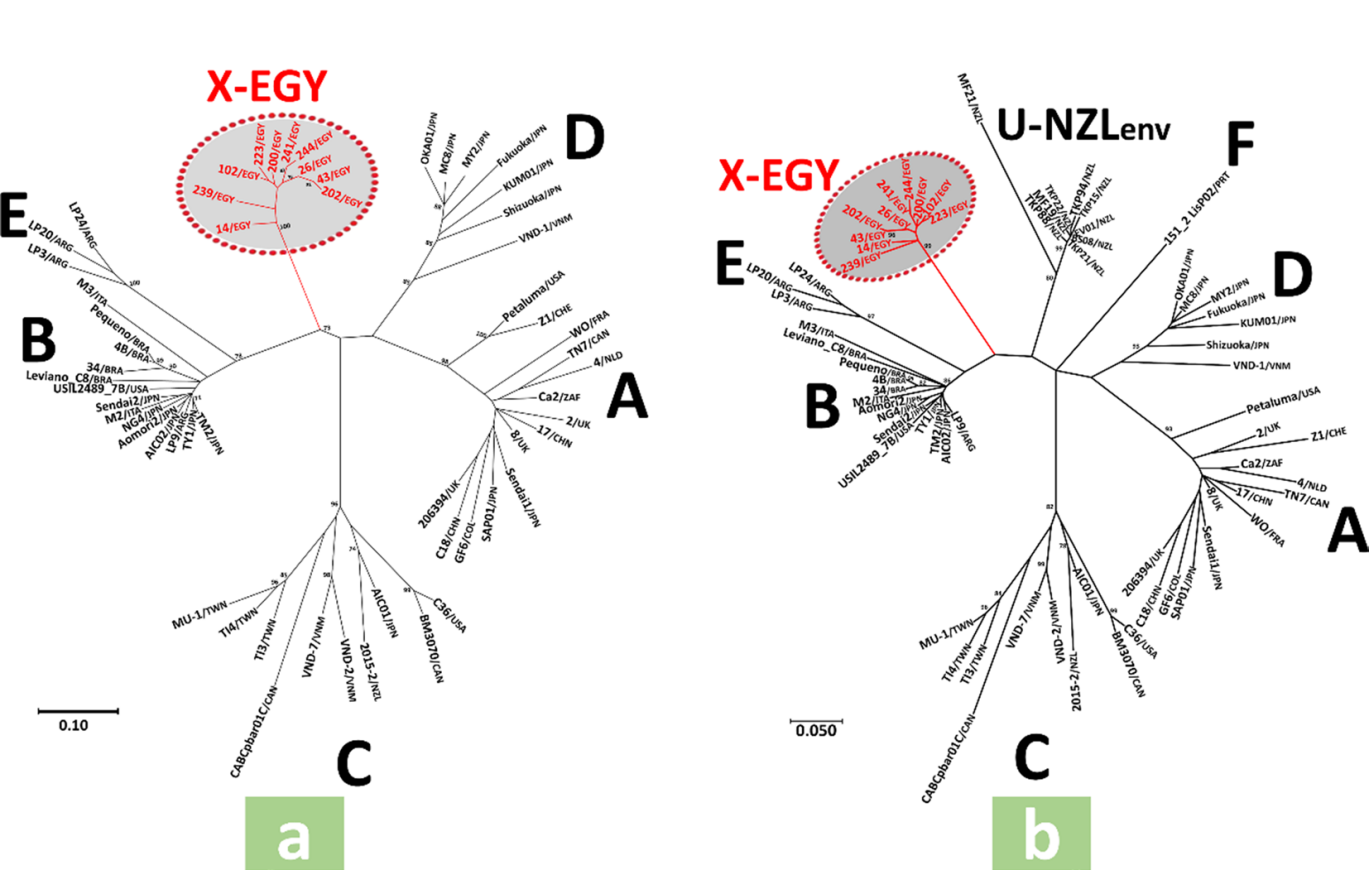
The *env* amino acid (aa) sequence-based phylogenetic analyses conducted using the maximum likelihood statistical method and JTT + I + G aa substitution model. The GenBank accession numbers of sequences used to construct these trees are given in Tables 5 and 6. Bootstrap values > 70% are displayed at branching nodes. Names of the previously characterized FIV subtypes are displayed next to corresponding clades. The Egyptian strains are marked by red font, and their previously uncharacterized clade, designated as “*X-EGY*”, is highlighted in grey; these 10 strains represent all samples that tested positive by end-point PCR targeting the *env*3–5 region among the 76 FIV-infected cats identified in this study (10/76, 13.2%). The scale bar represents the percent sequence diversity. (**a**) The tree was constructed from 58 FIV aa sequences encompassing the variable regions V3 to V5 of the *env* protein, and there were 207 positions in the final dataset; this tree did not include subtypes F and U-NZ*env* as their available *env* gene sequences in GenBank either lack the V5 region (subtype F) or contain a long sequence of ambiguous nucleotides (subtype U-NZ*env*). (**b**) The tree was constructed from 68 FIV aa sequences encompassing the variable regions V3 to V4 of the *env* protein, and there were 160 positions in the final dataset

**Fig. 3:**
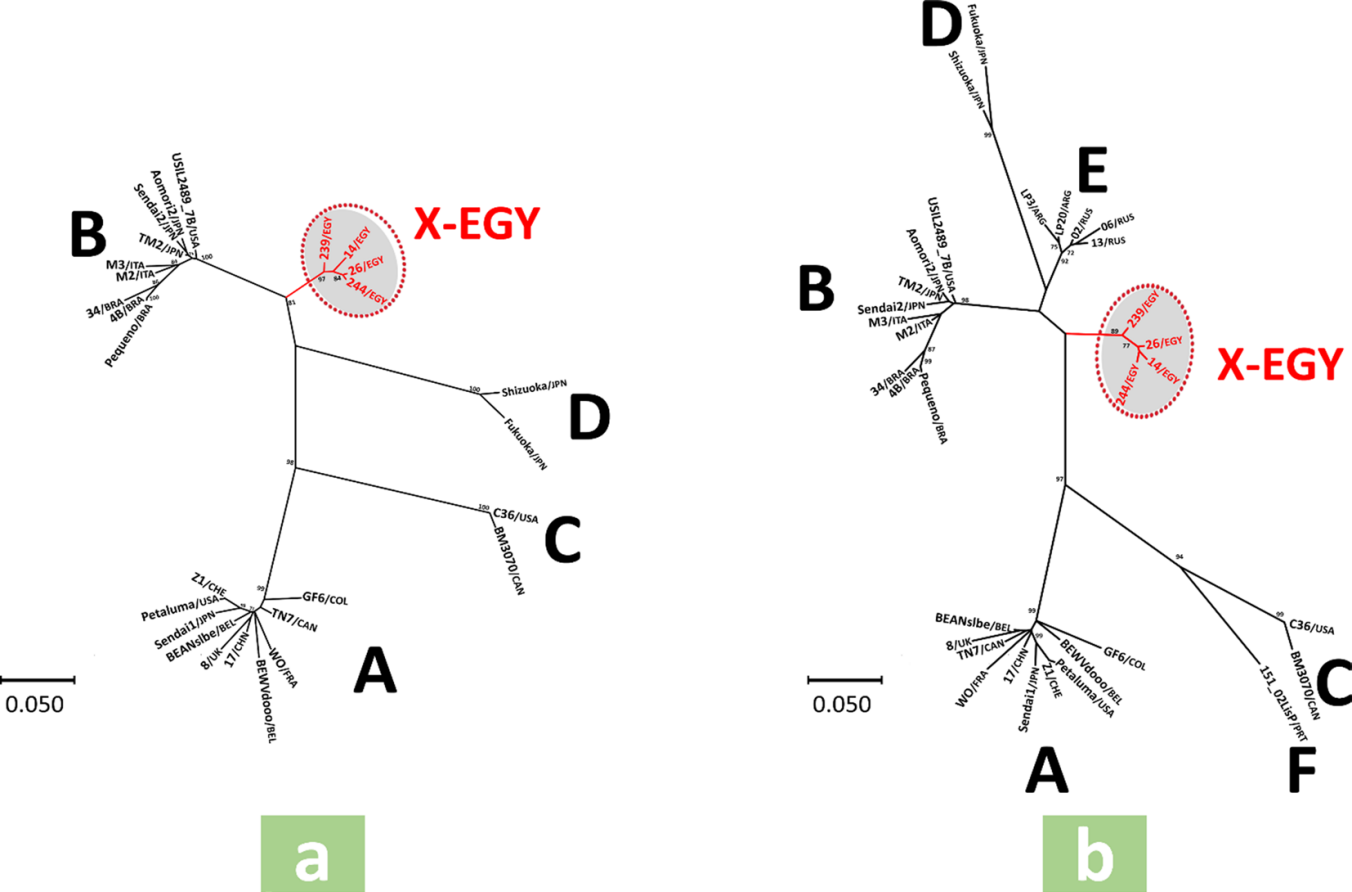
Phylogenetic trees of *gag* nucleotide alignments (**a**) 600 bp and (**b**) 300 bp, as inferred by the maximum likelihood statistical method and T92 + G nucleotide substitution model. The GenBank accession numbers of sequences used in this analysis are presented in Tables 4 and 5. Bootstrap values > 70% are displayed next to nodes. Names of the previously characterized FIV subtypes are shown next to the corresponding clade. The Egyptian strains identified in this study are marked in red font, and their newly characterized clade, designated “*X-EGY*”, is highlighted in grey; These include the four samples that tested positive by end-point PCR targeting the *gag* gene and were suitable for sequencing, out of the 10 *env*3–5 PCR-positive samples identified in this study (4/10, 40%). The scale bar indicates the genetic divergence percentage of sequences

The *env3-5* gene nt sequence-based ML tree showed six main clades; five contained strains of previously characterized FIV subtypes (A-E), while the sixth included only Egyptian strains obtained in this study, supported by a high bootstrap value (Fig. 1a). The *env3-4* gene nt sequence-based ML trees showed eight main clades; seven contained strains of previously characterized FIV subtypes (A-F and U-NZ*env*), while the eighth included only Egyptian strains obtained in this study, supported by a high bootstrap value (Fig. 1b). The same clades observed in the *env3-5* and *env3-4* gene nt sequence-based ML trees were obtained in their corresponding aa sequence-based ML phylogenetic trees (Fig. 2a and b). Similarly, in ML trees based on *gag* gene nt sequences, regardless of length (300 or 600 nt), Egyptian strains clustered separately into an independent clade, supported by high bootstrap values, distinct from other previously characterized subtype (A-F) clades (Fig. 3a and b). The average *env* nucleotide sequence divergence percentages within Egyptian strains were 3.7% and 2.8% for *env3-5* and *env3-4* gene segments, respectively, while the divergence between Egyptian strains and known FIV subtypes ranged from 21.2 to 24.9% for *env3-5* and 20.1–25.3% for *env3-4* gene segments (Table 7). No recombination events were detected in different gene segments of the Egyptian strains using all recombinant detection methods.

**Table 7:** Average percentages of genetic divergence among and between FIV subtypes as determined by Kimura-2-Parameter-pairwise genetic distance matrix for *env3-5* gene sequence (A), and *env3-4* gene sequence (B)

A									
Subtype	A	B	C	D	E	X-EGY			
A	8.6								
B	24.7	5.6							
C	26.5	23.1	12.2						
D	24.5	19.7	24.4	9.2					
E	24.5	18.9	24.7	20.9	4.6				
X-EGY	24.5	21.2	24.3	23.8	24.9	3.7			

B									
Subtype	A	B	C	D	E	F	U-NZ*env*	X-EGY	
A	7.6								
B	23.5	4.6							
C	24.3	20.9	10.5						
D	23.2	18.4	22.3	8.05					
E	23.3	17.3	22.4	20.0	4.01				
F	23.0	20.9	22.5	20.2	22.4	-			
U-NZ*env*	22.6	21.7	21.0	23.2	22.2	19.3	3.06		
X-EGY	24.1	20.1	23.2	23.5	22.8	22.4	25.3	2.8	

**Abbreviations** (K2P: Kimura-2-parameters model; U-NZ*env*: Unknown-New Zealand *env* subtype)

**Fig. 4:**
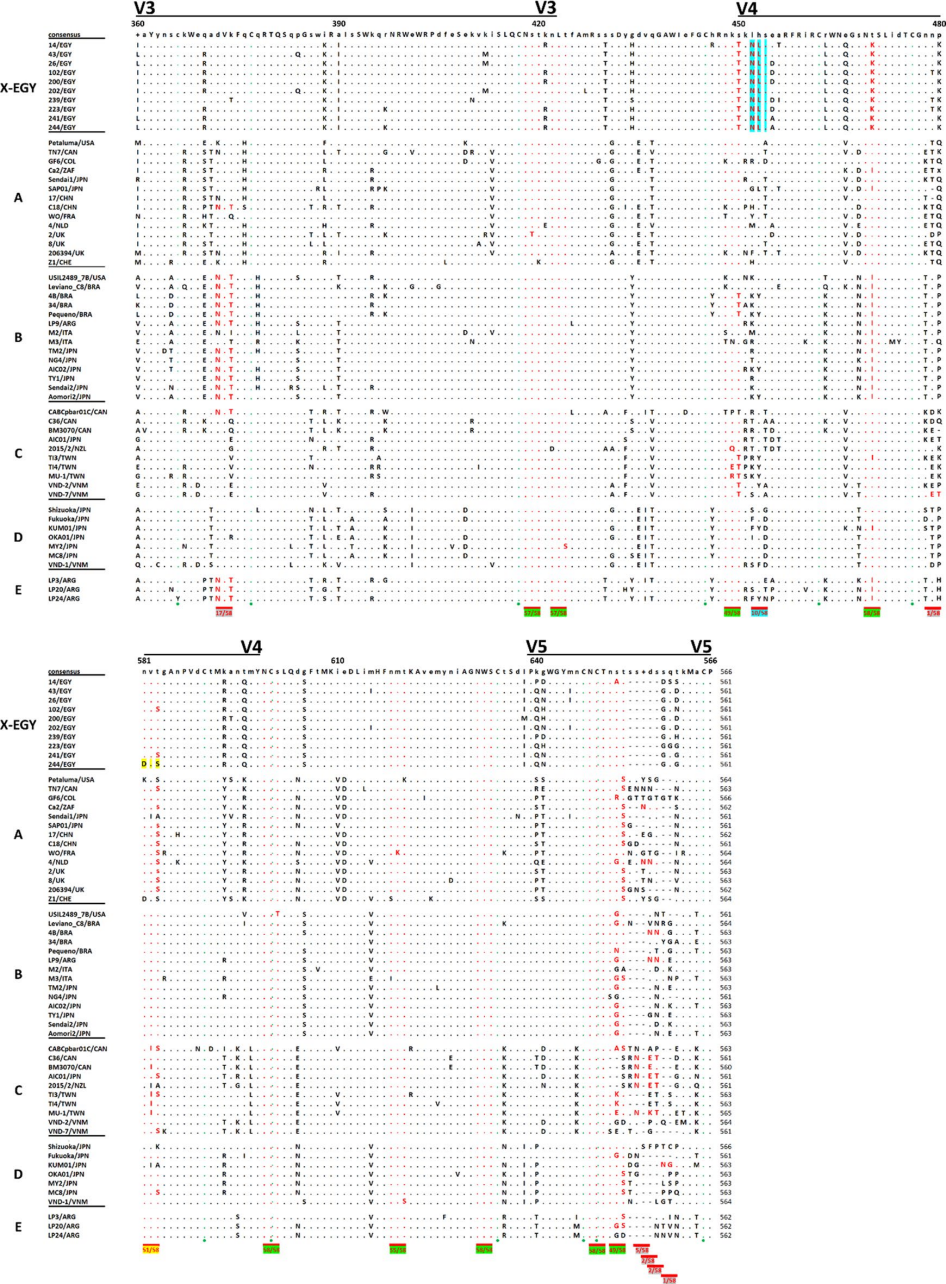
An alignment of predicted amino acid sequences of *env*3-5 nucleotide sequences used in this study (Tables 5 and 6). A consensus sequence was calculated (capital letters indicate 100% conservation of these amino acid residues among all sequences, small letters display the most commonly encountered amino acid in this position among sequences, and the symbol “+” points out positions with equal frequency of different amino acids among sequences). Positions identical to or different from the consensus sequences are shown in dots or amino acid letters, respectively; also, gaps are represented by dashes. This alignment involved three variable regions in the FIV *env* protein sequence (V3, V4, and V5) marked by black lines at the top of the alignment and separated with conserved regions. The numbers given at the top of the consensus sequence indicate the positions of amino acids according to the *env* protein sequence of the reference strain Petaluma/USA. Cysteine residues are marked by green circles under the alignment; a total of 12 cysteine residues were found, and all of them were conserved among all sequences except for one cysteine residue at position 366, which showed the mutation “C366Y” in FIV strain L24/ARG. Potential *N*-linked glycosylation sites are marked by red dashes under the alignment alongside the number of sequences displaying these sites. Potential *N*-linked glycosylation sites encountered in all Egyptian strains alongside other reference strains are highlighted in green, while one unique site presenting only in all Egyptian strains was highlighted in blue. Potential *N*-linked glycosylation sites absent from only one or all Egyptian strains are highlighted in yellow and grey, respectively. The position number of the last amino acid is given at the end of each sequence to indicate the length variability of this gene segment, commonly observed in the V5 region due to deletion mutations

Analysis of 58 *env3-5* aa sequences, including the 10 Egyptian sequences generated in this study and 48 reference sequences, showed perfect conservation of cysteine residues, with most predicted N-glycosylation sites well-conserved. However, some variations were observed, including a unique N-glycosylation site in all Egyptian strains (Fig. 4). The average *env* aa sequence divergence percentages within Egyptian strains were 4.8% and 3.7% for *env3-5* and *env3-4* aa sequences, respectively, while the divergence between Egyptian strains and known FIV subtypes ranged from 19.8 to 25.9% and 19.4–28.8% for *env3-5* and *env3-4* aa sequences, respectively (Table 8).

**Table 8:** Average percentages of divergence within and between FIV subtypes as determined by the Jones-Taylor-Thornton (JTT)-pairwise genetic distance matrix for *env3-5* (A) and *env3-4* (B) amino acid sequence

(A)								
	A	B	C	D	E	X-EGY		
A	12.2							
B	24.9	7.8						
C	25.4	24.6	15.2					
D	21.4	22.5	26.2	11.0				
E	25.9	17.4	25.7	21.9	8.4			
X-EGY	23.2	19.8	24.9	24.0	25.9	4.8		

(B)								
	A	B	C	D	E	F	U-NZ*env*	X-EGY
A	11.6							
B	28.6	6.6						
C	25.6	25.6	15.1					
D	22.5	24.1	27.4	9.7				
E	25.9	15.8	27.9	22.1	7.2			
F	27.6	27.1	28.1	25.1	27.5	-		
U-NZ*env*	26.1	19.6	23.8	22.1	20.1	23.9	5.1	
X-EGY	28.7	19.4	26.1	27.4	25.5	26.1	28.8	3.7

## Discussion

In the current study, to our knowledge, FIV prevalence was estimated for the first time in client-owned and shelter-housed cats in Egypt. To accurately ascertain the FIV infection status of the study population, we used a diagnostic panel rather than relying on a single diagnostic technique. Anigen^®^ and SNAP^®^ kits were specifically selected for this panel as they are the only commercially available kits in Egypt that have been previously evaluated in several independent studies, demonstrating high diagnostic accuracy [11, 18, 19]. Additionally, the *env3-5* PCR methodology designed in a previous study [23] was included in this panel since it has been shown to detect commonly circulating FIV subtypes in different geographic regions [22, 23, 27, 28]; however, its performance in identifying geographically restricted or highly divergent strains has not been established.

FIV-infected cats usually test positive with PoC kits since detectable titers of FIV-specific antibodies are typically present during all infection stages [11]. In contrast, they do not necessarily test positive by PCR, as proviral DNA loads are often low or fluctuate during the subclinical or even terminal phase. Furthermore, the high genetic diversity of FIV commonly interferes with PCR amplification of the virus [11, 14, 24]. Therefore, cats that test positive on two PoC kits should be considered FIV-infected regardless of their PCR results; this statement is true given that these cats are neither vaccinated nor below six months of age to exclude vaccine-induced and maternally derived antibodies as causes of PoC kit false-positive results [11]. In the current study, all cats tested positive on both Anigen^®^ and SNAP^®^ kits only (*n* = 66) were regarded as FIV-infected since the FIV vaccine is not commercially available in Egypt, and all sampled cats were older than six months.

For currently undetermined reasons, Anigen^®^ and SNAP^®^ kits may yield conflicting results in a few cats [18, 20]. In such cases, the true FIV infection status should be determined using an alternative diagnostic method, such as PCR or virus isolation [11]. In this study, only four cats exhibited this discrepancy, and all were classified as FIV-negative since their PCR results were negative.

Occasionally, FIV-infected cats may test positive by PCR but negative by PoC kits [24, 29]. This occurs because PCR can detect infected cats earlier than PoC kits (2–4 vs. 4–8 weeks post-infection). Additionally, in some infected cats entering the terminal phase, FIV-specific antibodies may decline to undetectable levels due to immunodeficiency [1]. In this case, cats should not be considered FIV-infected until the identity of the detected amplicons is confirmed as FIV by sequencing [24]. Only one FIV-infected cat (1/76, 1.3%) in this study exhibited this PCR/PoC kit disagreement, reflecting its rare occurrence. The result of its PCR was confirmed by sequencing. The cat was asymptomatic, suggesting it was in the acute rather than the terminal phase.

In the context of the pronounced *env* divergence observed at both nucleotide and protein levels among FIV strains in this study, the high sensitivity of sequential serological testing using SNAP^®^ and Anigen^®^ kits (98.7%) is not surprising. Both kits target antibodies against highly conserved viral proteins across subtypes [21]. The SNAP^®^ kit detects antibodies to p15 and p24, which are internal gag proteins shielded from immune pressure and structurally essential, explaining their high conservation. The Anigen^®^ kit targets antibodies against gp40, a transmembrane domain of the *env* protein that exhibits limited variability compared to the surface-exposed gp120, owing to its membrane-anchored position and critical function in viral fusion. Beyond the typically high antibody titers across all infection stages, this conserved antigen targeting underlies the robust diagnostic performance of both kits, even in the face of substantial viral diversity. This is supported by three independent studies demonstrating 100% or near-100% sensitivity in cats infected with diverse subtypes (A–F) [12, 18, 20]. Together with our findings, these studies provide indirect but compelling evidence of broad subtype coverage by the SNAP^®^ and Anigen^®^ kits, in light of the manufacturers not disclosing which subtypes were used in kit design and validation.

While PCR is known to have limited sensitivity for FIV detection in cats, with reported rates as low as 40% [11, 14, 18, 30], the PCR assay used in this study demonstrated a much lower sensitivity (10.3%). This is likely due to both the use of a highly variable target region (*env3–5*) and the lack of validation across all FIV subtypes, particularly geographically distinct or highly divergent strains such as the one identified here. These factors may have contributed to primer mismatches, which, together with the typically low proviral loads in infected cats, significantly reduced the likelihood of detecting FIV by PCR in most infected cats in this study. Importantly, all PCR-negative samples tested positive for the GAPDH internal control in a prior investigation [17], definitively ruling out extraction failure and affirming that the reduced sensitivity reflects a true performance limitation of the PCR assay itself. For future large-scale epidemiological studies relying solely on molecular detection, we strongly recommend using more sensitive PCR approaches, such as nested or real-time PCR targeting conserved regions like *gag*, ideally with primers designed based on locally circulating strains, if available. Positive samples can then undergo *env3–5* PCR for molecular characterization and subtype assignment. This two-step approach could improve both detection and genetic analysis of FIV strains [20, 31].

The FIV prevalence estimated in client-owned and shelter-housed cats in this study (31.7%) is slightly lower than that reported in feral cats in Egypt (33.9%) [15]. However, the prevalence observed in this study is considerably higher than that documented in most other regions worldwide, including African and Middle Eastern countries, as well as Asia, Europe, North America, South America, and Oceania [9].

The extremely high FIV prevalence in this study may be attributed to several factors: first, the substantial proportion of shelter-housed cats in the population. Recognizing FIV more frequently in shelter-housed cats has been consistently observed in FIV prevalence studies [8, 9]. The rationale behind such observations is that most shelter cats presumably had outdoor access in their life history, suggesting their engagement in fighting events with other cats, which is the major FIV transmission route. Additionally, shelter environments are characterized by overcrowding, high population turnover, and financial constraints that limit routine infectious disease testing, all of which facilitate infection transmission and may further explain the elevated FIV burden in this population [9]. In this study, FIV infection was significantly higher in shelter-housed cats compared to client-owned cats, and none of the participating shelters practiced retrovirus screening of cats before admission. Second, the exceptionally high FIV prevalence observed may be partly due to a genetically divergent strain with potentially enhanced infectivity, replication, or transmission, as seen in some divergent human immunodeficiency virus strains [32].

In this study, the FIV-infected group exhibited a decreased HCT%, albeit within the normal reference range, consistent with findings from a previous study [10] but contrasting those reported in other studies [26, 33]. This mild reduction in HCT% and the inconsistency among studies suggest that FIV plays a minor role in feline anemia. When anemia occurs, it is typically mild to moderate and arises primarily due to FIV-associated chronic disease rather than the virus’s direct affinity for infecting and destroying RBCs [10].

Regarding the leukogram, FIV-infected cats exhibited lymphopenia, as previously reported [10, 33, 34]. FIV is a lymphotropic virus, formerly designated as *T-lymphotropic lentivirus* [35], and it preferentially replicates within CD4^+^ T lymphocytes, leading to their depletion and dysfunction, the primary mechanism underlying FIV-induced immunodeficiency [11].

Although thrombocytosis was significantly associated with FIV infection in our study, this finding should be interpreted with caution. Thrombocytosis is not a commonly reported hematologic abnormality in FIV-infected cats; previous studies have more frequently documented thrombocytopenia or normal platelet counts [10, 26, 33, 34]. Notably, thrombocytosis was also observed more frequently than thrombocytopenia in FIV-non-infected cats, suggesting the involvement of shared contributing factors across both groups. Physiological thrombocytosis may result from catecholamine release during stress, leading to splenic contraction and transient platelet elevation [36]. Additionally, reactive thrombocytosis is a well-established response to chronic inflammation, often mediated by interleukin-6-induced thrombopoietin production by the liver [36, 37]. In our study, cats in both the FIV-infected and uninfected groups were likely exposed to stress during restraint and venipuncture, and many were housed in overcrowded shelter conditions that may contribute to physiological stress responses. Furthermore, a substantial proportion of both FIV-infected (35.5%) and uninfected (26.8%) cats exhibited chronic upper respiratory infection, potentially promoting sustained inflammatory signaling. While FIV is a chronic viral infection capable of modulating immune responses, the observed thrombocytosis is more plausibly attributed to a combination of shared physiological and pathological factors rather than to FIV infection alone.

Furthermore, FIV infection was associated with hyperglobulinemia and a subsequent decrease in the A: G ratio, a hallmark FIV-related serum biochemical abnormality consistently reported in previous studies, and attributed to the exaggerated, nonspecific, chronic polyclonal stimulation of B cells [10, 11, 26, 34, 38].

In the clinicopathological component of our study, no inclusion or exclusion criteria were applied. While this approach may limit our ability to attribute specific changes solely to FIV infection, it was chosen to reflect the natural variability of FIV infection as it presents in the field. By embracing this heterogeneity, our approach offers several practical advantages: first, it provides a comprehensive view of the clinicopathological spectrum that veterinarians are likely to encounter in FIV-positive cats; second, it enables the identification of diagnostic markers that remain consistent across diverse clinical presentations, which is particularly valuable in resource-limited settings; and third, it enhances the external validity of our findings, making them more generalizable and clinically relevant. From a statistical perspective, this design also facilitates the detection of robust abnormalities that might be diluted or overlooked in more narrowly defined cohorts.

The consistency of our key findings, particularly lymphocytopenia, hyperglobulinemia, and reduced A: G ratio, with those reported in previous case-control studies using strict inclusion criteria [10, 11, 26, 34, 38], reinforces the reliability of these abnormalities as indicators of FIV infection. While lymphocytopenia and thrombocytosis were the predominant hematological findings, some infected individuals exhibited lymphocytosis and thrombocytopenia. Such variation, also reported in more controlled studies [26, 33, 34], likely reflects the complex interplay of factors in naturally infected populations, including infection stage, immune response variability, and concurrent conditions such as FeLV, which are inherently difficult to fully control.

To our knowledge, this study is the first to provide a molecular characterization of FIV in Egypt, delivering essential baseline data on the genetic composition of circulating strains. The *env*3-5 gene region has long been recognized as a highly informative segment for FIV molecular epidemiology worldwide, due to its considerable genetic diversity and subtype-specific variation [2, 3, 5, 11, 22, 27, 28, 39–42]. Indeed, phylogenetic analyses of the env3-5 fragment have historically led to recognizing new FIV subtypes, including subtypes C [6] and E [43]. To ensure the accuracy of our phylogenetic inference, we excluded subtype F and U-NZ*env* sequences from the *env*3-5 analysis because subtype F lacks the complete V5 region, and U-NZ*env* sequences contain extensive ambiguous nucleotides, which could compromise alignment quality and tree robustness. Since previous studies have also utilized the shorter *env*3-4 segment for subtype determination [7], we conducted parallel phylogenetic analyses using both env3-5 and *env*3-4 nucleotide and amino acid sequences to comprehensively assess the genetic relationships of Egyptian strains relative to all known subtypes. The Egyptian strains clustered together in a distinct, previously uncharacterized clade, clearly divergent from all recognized subtypes (A-F and U-NZ*env*) (Figs. 1 and 2). This divergence is further supported by average genetic distance values within the expected intra- and inter-subtype diversity ranges reported in the literature [4, 6]. Although comparable data for aa sequence diversity are limited, the pronounced differences observed at the protein level (Table 8) reinforce the nucleotide-based phylogenetic findings and suggest potential structural and antigenic novelty in the env glycoprotein of these Egyptian strains.

To further elucidate the phylogenetic relationships of the Egyptian strains, we applied an additional PCR targeting the *gag* gene to all *env* PCR-positive samples, successfully obtaining sequences from four samples. Because available *gag* gene reference sequences in GenBank corresponding to our strains’ genomic region (~ 600 nt) were limited to a few subtypes (A–D), we also performed phylogenetic analyses using a shorter *gag* gene fragment (~ 300 nt) to incorporate a broader range of subtypes (A–F). Both the longer (~ 600 nt) and shorter (~ 300 nt) *gag* gene sequences consistently clustered within a distinct, previously uncharacterized clade, reinforcing the genetic divergence of the Egyptian strains (Fig. 3).

Importantly, recombination analyses revealed no evidence of recombination within either the *env* or *gag* sequences, excluding recombination as a driver of the observed high genetic divergence. Epidemiologically, the *env* and *gag* gene sequences originated from cats inhabiting geographically diverse locations across three Egyptian governorates, representing both client-owned and shelter-resident populations, and were collected over an extended period (January 2023-April 2024) (Table 5). This widespread detection strongly supports that this highly divergent strain is broadly circulating within Egyptian cats.

Taken together, the consistent phylogenetic clustering across two independent genomic regions, the absence of recombination, and the epidemiological evidence support the designation of these strains as a novel FIV subtype, tentatively named “*X-EGY*”. Notably, the absence of other known FIV subtypes and the low intra-subtype genetic variability observed in both the highly variable *env* gene and the more conserved *gag* gene suggest that this clade is the dominant circulating strain and likely represents an ancient lineage uniquely adapted to the Egyptian feline population rather than a recently emerged variant.

As expected, all 58 *env3-5* aa sequences exhibited complete conservation of cysteine residues, which play a critical role in maintaining the structural integrity of the viral glycoprotein. Interestingly, the unique *N*-glycosylation site observed in all Egyptian strains could be linked to the high FIV prevalence estimated in Egypt, as changes in *N*-linked glycosylation sites have been shown to positively or negatively alter viral infectivity, transmissibility, and ability to evade the immune System [3, 44].

The first limitation of this study is the small number of client-owned cats compared to shelter-housed cats, likely due to the perception among cat owners that their indoor cats are at a lower risk of FIV, which may have led to a reluctance to participate in the study. This discrepancy between shelter-housed and client-owned cats may have resulted in an inflated FIV prevalence in the study population and could potentially distort the statistical analysis.

The second limitation of this study is the relatively small size of the sequences analyzed, which is insufficient to support the development of a subtype-specific PCR assay essential for epidemiological surveillance and pathogenesis studies of this novel subtype. Therefore, further research focusing on full-genome sequencing is strongly encouraged to enable more comprehensive characterization of this divergent strain and to facilitate the design of targeted epidemiological tools.

## Conclusions

The present study provides valuable insights into FIV’s epidemiology and molecular evolution in Egypt, a country with limited data in this field. Employing a diagnostic panel rather than a single diagnostic technique likely enabled a more reliable estimation of FIV prevalence by incorporating screening and confirmation steps, effectively addressing conflicting results, and detecting infected cats at different infection stages. The findings underscore the limitations of molecular diagnosis compared to sequential serological methods, particularly in regions where the molecular characterization of locally circulating strains has not yet been conducted. Additionally, Egypt continues to record one of the highest FIV prevalence rates worldwide. The phylogenetic analysis revealed that Egyptian FIV strains are highly divergent from all previously characterized subtypes, suggesting the existence of a new subtype, *X-EGY*. The low genetic divergence of viruses constituting this new clade, alongside its widespread circulation in Egyptian cats, precludes the possibility of its recent emergence. Similar to this study, ongoing FIV molecular characterization studies of FIV, particularly in geographic areas with limited data, are crucial for improving our understanding of the evolution of this genetically unstable virus.

## Data Availability

DNA sequences generated in this study were submitted to GenBank (NCBI) following accession: OR863244, OR882079-OR882081, and PQ799455-PQ799464.
